# Toward common treatment strategies: convergent proteinopathies and mitochondrial dysfunction in Alzheimer’s and Parkinson’s diseases

**DOI:** 10.3389/fnins.2026.1846384

**Published:** 2026-05-26

**Authors:** Julie E. V. Offerdahl, Danielle E. Mor

**Affiliations:** Department of Neuroscience and Regenerative Medicine, Medical College of Georgia at Augusta University, Augusta, GA, United States

**Keywords:** Alzheimer’s disease, mitochondria, Parkinson’s disease, tau, α-synuclein

## Abstract

Alzheimer’s disease (AD) and Parkinson’s disease (PD) are the two most prevalent neurodegenerative disorders (ND) globally, disproportionately affecting the elderly population. Traditionally viewed as distinct diseases, AD is defined by symptoms of cognitive impairment and dementia with amyloid-β and tau protein pathologies, while PD is defined by motor symptoms and eventual dementia with α-synuclein (α-syn) protein pathology. However, these pathologies are not unique to either disease, with a large fraction of AD patients displaying α-syn inclusions and PD patients displaying abnormal tau. Emerging evidence indicates that pathological tau and α-syn not only frequently coexist in AD and PD, but may engage in synergistic interactions that promote mitochondrial dysfunction, accelerate neurodegeneration, and worsen cognitive decline in both disorders. This review aims to provide both the prevailing views of AD and PD, as well as a detailed discussion of their commonalities with a focus on how tau and α-syn toxicities intersect at the mitochondrial level. Common features of mitochondrial impairment in AD and PD are discussed, including complex I deficiency, oxidative stress, impaired axonal transport, altered mitochondrial dynamics, and mitochondrial DNA damage. While prior reviews have often examined AD and PD independently, this review specifically focuses on the convergent and potentially synergistic interactions between tau and α-syn at the level of mitochondrial dysfunction, highlighting a shared mechanistic framework that may inform unified therapeutic strategies. By studying and understanding the mutual mechanisms underlying neurodegeneration in AD and PD, common treatment strategies can be identified.

## Highlights

- Alzheimer’s and Parkinson’s diseases are the most common neurodegenerative disorders.
- Cognitive decline and dementia occur in both Alzheimer’s and Parkinson’s diseases.
- Overlapping disease mechanisms, particularly related to mitochondrial dysfunction, may unlock shared treatment strategies.

## 1 Introduction

### 1.1 Alzheimer’s disease

First documented by Alois Alzheimer in 1906, Alzheimer’s disease (AD) is the leading neurodegenerative disorder and the most common form of dementia, accounting for up to 80% of all dementia cases (Hippius and Neundörfer, 2003; Nussbaum and Ellis, 2003; Barnes and Yaffe, 2011). Aging is the greatest risk factor for the development of AD, with an estimated 6.9 million individuals aged 65 years and older living with AD in the United States alone, and over 55 million people afflicted with AD worldwide (Hou et al., 2019; Leuner et al., 2012; Alzheimer’s Disease International, 2020). The majority of AD cases are sporadic (90–95%), generally occurring after the age of 65, while 5–10% are familial and occur earlier in life due to hereditary genetic mutations (Piaceri et al., 2013).

AD is characterized by progressive memory impairment and cognitive decline, affecting both declarative and non-declarative memory, including the recall of facts and events as well as learning capacity (Squire and Zola, 1996; John and Reddy, 2021). As the disease progresses, broader cognitive and functional impairments emerge, including deficits in reasoning, language, and visuospatial processing, often accompanied by behavioral and psychological symptoms such as anxiety, depression, agitation, and sleep disturbances (Alzheimer’s Association, 2022).

AD is a complex and progressive disease with underlying mechanisms and pathologies that often take place years before the onset of clinical symptoms, only clinically addressed once mild cognitive impairment is present. By the time mild cognitive impairment becomes apparent and a diagnosis is made, significant neuronal dysfunction has already occurred. Although current treatments cannot cure AD, they may modestly slow symptom progression.

### 1.2 Parkinson’s disease

Originally called the “shaking palsy” upon discovery and documentation by James Parkinson in 1817, Parkinson’s disease (PD) is the second most common neurodegenerative disease, classically presenting with motor symptoms (Parkinson, 2002; Tran et al., 2020). Global estimates from 2019 showed over 8.5 million individuals living with PD (Parkinson’s Foundation, 2025). PD is an age-related disease with onset generally occurring around 60 years of age or later (Reeve et al., 2014; Tran et al., 2020). The sporadic form of PD constitutes 85–90% of cases while the remaining 10–15% are familial, caused by hereditary genetic mutations (Parkinson’s Foundation, 2025).

Symptoms of PD manifest as motor and non-motor symptoms, with motor signs being the hallmark presentation for diagnosis (Williams-Gray et al., 2007). Motor symptoms include tremors, muscle stiffness, bradykinesia, and postural instability. A variety of non-motor symptoms can often precede motor signs and a PD diagnosis by 10 years or more (Sveinbjornsdottir, 2016). These symptoms include gastrointestinal dysfunction, sleep disturbances, hyposmia, depression, and anxiety (Chen et al., 2013). However, other non-motor symptoms, such as cognitive impairment, may be present during the diagnosis of PD but may become more prominent in later stages (Gatt et al., 2016; Aarsland et al., 2021; Sveinbjornsdottir, 2016; Chen et al., 2013; Fang et al., 2020).

### 1.3 Overlap

AD and PD share significant clinical overlap, particularly in their age of onset and progression to cognitive impairment. Both disorders predominantly affect individuals over the age of 60 and are associated with the development of dementia (Gatt et al., 2016). While AD is the most common cause of dementia, cognitive decline is also highly prevalent in PD, affecting up to 30% of patients in early stages and up to 90% in advanced disease (Gatt et al., 2016; Aarsland et al., 2017; Collins and Williams-Gray, 2016). Consistent with this, individuals with PD have a 3.5–6.1-fold increased risk of developing dementia compared to the general population (Åström et al., 2022). Often, clinicians are unable to accurately diagnose whether a PD patient has developed Parkinson’s disease dementia or AD due to the similarities of disease presentation (Garn et al., 2017). Similarly, motor impairments, including tremor and dystonia, may emerge in later stages of AD, further highlighting the clinical convergence between these disorders (Andrade-Guerrero et al., 2024).

## 2 Neurodegeneration

### 2.1 Alzheimer’s disease

AD is characterized by progressive cerebral atrophy (Whitwell, 2010). Early in disease development, mild cognitive impairment occurs due to neurodegeneration in the entorhinal cortex and hippocampus regions essential for learning and memory (Scahill et al., 2002; Whitwell, 2010). Neuropathological studies indicate that the highest rate of neurodegeneration occurs in moderate to moderately severe stages, reaching a “critical point” before plateauing in the advanced disease (Wegiel et al., 2021; Jack et al., 2008). Neurodegeneration spreads from the medial temporal lobes and fusiform gyrus to the posterior temporal and parietal lobes (Whitwell et al., 2007). AD-related brain volume loss has been detected via MRI which provides visualization of widening sulci and shrinking gyri in the brain (John and Reddy, 2021). Autopsy findings have revealed the size of sulci may be reduced by up to 50% (Goel et al., 2022).

Multiple neuronal subtypes degenerate in AD. Degeneration of cholinergic neurons originating in the nucleus basalis of Meynert (nbM) within the basal forebrain (BF) leads to impairment in attention, learning, and memory (Whitehouse et al., 1981; Francis, 2005). The BF is the main source of cholinergic innervations to the neocortex and amygdala. As a result of BF degeneration, these regions show significant loss of cholinergic innervation. Glutamatergic neurons also undergo degeneration, accounting for a large proportion of neuronal loss in the hippocampus and cerebral cortex (Francis, 2005). Approximately two thirds of synapses in the hippocampus and neocortex use glutamate, supporting normal cognitive function (Fonnum, 1984). Dysfunctional glutamatergic neurons can result in reduced N-methyl-D-aspartate (NMDA) receptors, which are essential in learning and memory (Greenamyre et al., 1988). Though other neuronal subtypes are also involved in the pathogenesis of AD, evidence largely links cholinergic and glutamatergic neurons to AD-related cognitive impairments and decline.

### 2.2 Parkinson’s disease

There is strong evidence that neurodegeneration in PD begins in preclinical stages (Tremblay et al., 2021; Uribe et al., 2018). The most heavily studied area of the brain that undergoes degeneration in PD is the dopamine-producing substantia nigra (SN), a subregion of the basal ganglia within the ventral midbrain. When dopamine (DA) production decreases due to SN degeneration, the striatum and other regions of the basal ganglia undergo dopaminergic denervation (Blandini et al., 2000). The loss of basal ganglia circuitry function underlies the classic parkinsonian motor signs of PD, including tremors, postural instability, bradykinesia, and muscle rigidity.

Beyond the SN, degeneration is also observed in brainstem regions, including the locus coeruleus, raphe nuclei, and dorsal motor nucleus of the vagus, as well as cortical regions such as the temporal, parietal, occipital, and cingulate cortices (Filippi et al., 2020; Uribe et al., 2018). The thalamus, cerebellum, sensorimotor cortex, and cortical association areas are also affected (Li et al., 2022). Overall cortical atrophy has been associated with cognitive decline in PD, with evidence of thinning occurring even at early disease stages (Uribe et al., 2018; Pereira et al., 2014).

Degeneration of these regions is accompanied by loss of multiple neuronal subtypes, contributing to non-motor symptoms of PD. Cholinergic degeneration, specifically in the BF, is a central feature in the development of non-motor symptoms, most prominently cognitive decline. Degeneration and dysfunction of the serotonergic (5HT) system is implicated in the development of symptoms involving sleep disorders, cognitive dysfunction, depression and anxiety (Khowdiary et al., 2025). Similarly, loss of norepinephrine, primarily originating from the locus coeruleus, is associated with sleep disturbances, as well as cognitive impairment, depression, and anxiety (Del Tredici and Braak, 2013).

### 2.3 Overlap

Increasing evidence shows that affected regions in both AD and PD form interconnected networks throughout the brain (Caligiore et al., 2022). Cholinergic, glutamatergic, noradrenergic, and serotonergic pathways are affected in both diseases. One of the common regions of degeneration between AD and PD is the BF. The BF houses the densest population of cholinergic neurons in the brain which are heavily involved in learning, memory, attention, and emotional regulation (Geula et al., 2021). These neurons normally project to the neocortex, hippocampus, and amygdala. Cholinergic neurons of the BF are impacted early in the development and progression of both AD and PD. Interestingly, degeneration of the BF has been found to precede cognitive decline and may serve as a predictive marker for memory impairment in models of AD and PD (Schmitz et al., 2016).

AD and PD also share the impairment of monoamine neurotransmitter systems early on (Caligiore et al., 2022). Noradrenergic pathways of the locus coeruleus, which regulate memory and attention, are severely impaired in AD and PD (Giorgi et al., 2020). In fact, the locus coeruleus is one of the earliest regions to begin to degenerate in both diseases. 5HT pathways are impacted early on as well (Smith et al., 2017). Postmortem studies of AD patients found that 5HT receptor density was decreased in various locations of the brain (Solas et al., 2021; Hasselbalch et al., 2008). Strikingly, this reduction in receptor density has been associated with decreased striatal dopamine (DA) levels, resembling dopaminergic loss observed in PD (Smith et al., 2017).

## 3 Protein pathologies

### 3.1 Alzheimer’s disease

AD pathology centers around the extracellular accumulation of amyloid-beta (Aβ) plaques and intracellular neurofibrillary tangles (NFTs) composed of hyperphosphorylated tau (2024 Alzheimer’s disease facts and figures, 2024). Aβ is derived from the proteolytic cleavage of the glycoprotein amyloid precursor protein (APP), encoded by the *APP* gene (Tcw and Goate, 2017). APP contains three domains: a large extracellular region, a hydrophobic transmembrane domain, and a short C-terminus (Zhang et al., 2012). Full length APP has been proposed to function as a cell surface receptor via its extracellular domain (Zheng and Koo, 2011). Cleavage of APP by β-secretase generates insoluble Aβ monomers that are released into the extracellular space, where they self-associate into β-sheet–rich oligomers and eventually form plaques (Kurkinen et al., 2023; Zhaliazka and Kurouski, 2022). Aβ exists in multiple isoforms, the most common in AD being Aβ40 and Aβ42, with the only structural difference being two additional C-terminal residues (Itoh et al., 2022). This process can begin as early as 20 years before the onset of cognitive decline during a preclinical stage (Chun, 2018; Braak and Del Tredici, 2015). The preclinical stage begins with diffuse Aβ accumulation in cortical regions and progresses into allocortical regions and midbrain, ultimately reaching the cerebellum and brainstem during the clinical phase of AD (Hampel et al., 2021). By the time of diagnosis, Aβ plaques are densely spread throughout the brain. However, the plaque burden does not directly correlate with cognitive decline and dementia (Weigand et al., 2020). In contrast, tau pathology more closely coincides with the onset of cognitive impairment (Frontzkowski et al., 2022; Chu et al., 2025).

Tau is a microtubule associated protein located within neurons throughout the central nervous system (Goedert et al., 1988). In healthy cells, tau supports microtubule assembly, stabilization, and axonal transport. Tau is encoded by the gene microtubule associated protein tau (MAPT) (Leveille et al., 2021). MAPT has two haplotypes, H1 and H2, with H1 associated with increased expression and neurodegeneration, while H2 may be protective against certain tauopathies (Leveille et al., 2021; Webb et al., 2008). In patients exhibiting the H1 haplotype, elevated levels of *MAPT* expression can also influence several other surrounding genes (De Jong et al., 2012). Regardless of haplotype, four major domains make up the protein tau: the N-terminus, the proline-rich region, the microtubule binding domain, and the C-terminus (Leveille et al., 2021). Six isoforms of tau exist, which differ based on the presence or absence of exons 2, 3, and 10 (Goedert, 2004). The number of N-terminal repeats is determined by exons 2 and 3 (0N, 1N, or 2N). The presence or absence of exon 10 is what determines whether tau contains 3 (3R) or 4 (4R) microtubule binding domain repeats. Normal adult brains express similar (1:1) levels of the 3R and 4R isoforms (Goedert, 2004).

In AD as well as other neurodegenerative diseases, the 3R:4R ratio of tau becomes disrupted (Chen et al., 2010). Specifically, in AD, 4R is increased and 3R is decreased resulting in an approximately 2:1 4R:3R ratio (Chen et al., 2010). Under normal conditions, tau is soluble and natively unfolded (King et al., 2000). When tau becomes hyperphosphorylated, it loses its affinity for microtubules and aggregates into β-sheet–rich filaments, forming NFTs through recruitment and misfolding of native tau (Congdon et al., 2008). Given that tau contains 85 potential sites for phosphorylation, it appears to be easily phosphorylated, with hyperphosphorylation occurring at certain epitopes linked with neurodegeneration, cognitive impairment, and dementia (Ballatore et al., 2007; Noble et al., 2013; Noble et al., 2011). Phosphorylation at Ser202/Thr205, Thr181, Thr217, Thr231, Ser356, Ser396, and Ser422 are most strongly associated with AD pathology and cognitive decline (Gobom et al., 2022). This abnormal phosphorylation may be due to upregulated protein kinase activity or downregulated protein phosphatase activity (Noble et al., 2011).

The tau 4R isoform is the most frequently identified isoform in AD and appears to aggregate more readily than other isoforms in AD (Buchholz and Zempel, 2024; Waheed et al., 2023). The entorhinal cortex is the first region to be impacted by tau pathology, with spreading to synaptically interconnected regions from there. Braak and Braak (1991) developed a staging scheme consisting of six stages for the development and progression of NFT pathology in AD. Stages I-II develop lesions in the transentorhinal and entorhinal regions, in stages III-IV lesions have expanded into the limbic allocortex and adjoining neocortex, and lastly in stages V-VI the pathology extends widely throughout the neocortex (Braak and Braak, 1991). This patterned distribution of NFTs parallels neuronal loss and brain atrophy previously discussed (DeTure and Dickson, 2019).

### 3.2 Parkinson’s disease

The pathological hallmark of PD is the presence of Lewy bodies (LBs) and Lewy neurites (LNs), which are primarily composed of α-syn (Goedert et al., 2013; Mehra et al., 2019). The physiological role of α-syn is still under investigation; however, it is known that α-syn is a small intracellular protein that is abundantly expressed in neurons, localizing to presynaptic terminals (Villar-Piqué et al., 2016). The expression of α-syn is encoded by the gene *SNCA* which is located at position 21 in the long arm of chromosome 4. Its primary amino acid sequence can be divided into three major domains: the N-terminal domain containing amphipathic α-helices that allow for membrane binding, the central domain which is known as the non-amyloid-beta component of Alzheimer’s disease amyloid (NAC) domain, and the C-terminal domain that is largely unstructured and mediates protein-protein interactions and metal binding (Stefanis, 2012; Culvenor et al., 1999). Interestingly, the NAC domain of α-syn was first identified as a constituent of amyloid plaques in AD patients (Uéda et al., 1993).

The NAC domain is highly hydrophobic and essential for α-syn aggregation (Culvenor et al., 1999). Natively, α-syn is unfolded yet compact due to the interactions between the NAC domain and C-terminus, preventing aggregation (Negi et al., 2024). Disturbances in α-syn compactness are thought to allow α-syn to misfold, leading to aggregation and the formation of intracellular inclusions (Villar-Piqué et al., 2016). α-Syn aggregation also follows the amyloidogenic pathway whereby soluble oligomers convert to insoluble fibrils with distinct cross β-sheet conformation. These aggregates can template α-syn monomers in a prion-like manner, amplifying aggregation (Villar-Piqué et al., 2016; Pan et al., 2022). In autosomal dominant PD, there are mutations in *SNCA* which enhance or modify the aggregation properties of α-syn. For example, the A53T mutation accelerates α-syn fibrillization (Villar-Piqué et al., 2016). While the sporadic form of PD lacks known mutations in *SNCA* or other genes (Spillantini et al., 1997, Chiba-Falek et al., 2006), extensive studies have shown that wild type α-syn inclusions develop and appear to spread throughout the nervous system.

Similar to tau in AD, Braak et al. (2003) developed a staging system consisting of six stages to describe the progression of α-syn pathology in the brain in PD. In the first stage, α-syn lesions develop in the medulla oblongata, then in the pontine tegmentum during stage 2 (Braak et al., 2003). α-Syn pathology reaches the midbrain during stage 3, which includes the SN where dopaminergic degeneration occurs. Stage 4 progresses into the basal prosencephalon and mesocortex, but does not yet affect the neocortex. The final stages (stages 5 and 6) are denoted by the spread of lesions into the neocortex, damaging high order sensory association areas, eventually involving wide swaths of the neocortex (Braak et al., 2003; Goedert et al., 2013). More recently, α-syn pathology has also been described in the enteric nervous system in PD patients, with growing evidence suggesting that peripheral α-syn pathology forms prior to the appearance of Lewy pathology in the central nervous system (Chen and Mor, 2023). α-Syn pathology potentially originating in the gut aligns with the high occurrence of gastrointestinal dysfunction in early PD (Fasano et al., 2015).

### 3.3 Overlap

In AD and PD, as well as other neurodegenerative diseases, patients rarely present with a single protein pathology, but more commonly exhibit multiple proteinopathies, especially in late-onset forms of disease (Tosun et al., 2024; Ramirez et al., 2024; Robinson et al., 2023). Emerging evidence suggests that tau and α-syn have complex interplay in both AD and PD contexts (Lu et al., 2020; Fischer et al., 2025). Strikingly, α-syn pathology has been found in up to 60% of AD brains, while tau is present in up to 50% of postmortem PD brains, as well as in the related disorder, dementia with Lewy Bodies (Caligiore et al., 2022; Pan et al., 2022; Fontela et al., 2017; Guo et al., 2013; Giasson et al., 2003a; Moussaud et al., 2014; Kovacs et al., 2008; Higashi et al., 2007; Lippa et al., 1998; Irwin et al., 2013). Tau and α-syn have even been documented aggregated together within the same tangles in PD (Ishizawa et al., 2003; Arima et al., 1999; Savica et al., 2019; Moussaud et al., 2014). The presence of multiple protein pathologies, particularly tau and α-syn co-pathology, has been associated with accelerated clinical progression, including cognitive decline, and overall more than 50% of dementia patients exhibit mixed protein pathologies (Bellomo et al., 2024; Swirski et al., 2014; Kovacs et al., 2008; Shim et al., 2022; Compta et al., 2011; Clinton et al., 2010). Combined pathologies are a stronger predictor of cognitive decline and dementia than single pathologies, regardless of the severity of the single proteopathies (Compta et al., 2011; Sengupta and Kayed, 2022). Moreover, in PD patients with AD-like pathology, α-syn aggregation is significantly increased compared to PD patients without AD-like pathologies (Pan et al., 2022; Masliah et al., 2005). However, the precise mechanisms by which tau and α-syn co-pathologies accelerate cognitive decline remain unclear.

Given evidence of enhanced disease progression when both tau and α-syn are present, these proteins are hypothesized to interact synergistically (Caligiore et al., 2022). Previous reports have suggested that α-syn and tau can promote the fibrillization and insolubility of one another *in vitro* and *in vivo*, suggesting synergistic interactions and/or crosstalk (Williams et al., 2020; Frey et al., 2023; Bassil et al., 2021; Waxman and Giasson, 2011). In particular, α-syn has been found to promote the hyperphosphorylation of tau and initiate tau amyloid formation, while tau has been found to accelerate α-syn aggregation and spreading (Franzmeier et al., 2025; Jensen et al., 1999; Pan et al., 2022; Badiola et al., 2011; Lee et al., 2004; Giasson et al., 2003b). It has been documented that tau and α-syn induce cross-seeding of one another and that this may occur through prion-like mechanisms (Morales et al., 2013; Jeyabalan et al., 2025; Padilla-Godínez et al., 2025). Studies have even suggested that α-syn may be a contributor to the conformational change tau undergoes to create insoluble fibrils (Lee et al., 2004).

Overall, tau and α-syn share several key biochemical and pathological properties (Jeyabalan et al., 2025). Both proteins are intrinsically disordered in their native state but can form amyloid aggregates under disease conditions (Vacchi et al., 2020; Shim et al., 2022), adopting abnormal biochemical states, including hyperphosphorylation (Hasegawa et al., 2002). A further similarity that can promote tau and α-syn interactions is the fact that both proteins are localized intracellularly, whereas Aβ is extracellular.

## 4 Mitochondrial dysfunction

### 4.1 Alzheimer’s disease

Extensive studies implicate mitochondrial dysfunction as a central pathological feature of AD (Lin and Beal, 2006; Terada et al., 2021; Wang et al., 2020; Kerr et al., 2017; Hauptmann et al., 2006; Manczak and Reddy, 2012; Cai and Jeong, 2020; Pradeepkiran and Reddy, 2020; Tönnies and Trushina, 2017; Monzio Compagnoni et al., 2020; Reddy et al., 2012; Hirai et al., 2001; Reddy and Oliver, 2019; Yapa et al., 2021; Calkins et al., 2011). In AD brains, mitochondria have been found to have an array of abnormalities which often correspond with the presence of tau or Aβ (Reddy and Beal, 2008). Mitochondrial dysfunction in AD is generally evidenced by a reduction in enzymatic activity of cellular/mitochondrial respiration, altered ATP production, and by increased levels of free radicals and reactive oxygen species (ROS) which in turn may lead to mutations in mitochondrial DNA (mtDNA) (Hirai et al., 2001; Area-Gomez et al., 2018; Wang et al., 2025). In addition to, and potentially contributing to these abnormalities, there is documented decrease in the activity of mitochondrial complexes (MC) I-IV in both post-mortem AD samples as well as in cellular models (Gao et al., 2025; Terada et al., 2021; Terada et al., 2022; Adlimoghaddam et al., 2019; Troutwine et al., 2022). Altered mitochondrial complex I levels contributes to the loss of neuronal homeostasis in early AD pathology (Adlimoghaddam et al., 2019; Terada et al., 2021). Increased tau load and mitochondrial dysfunction, particularly involving complex I, are associated with memory decline in early AD (Terada et al., 2021).

Tau accumulation has been associated with abnormal mitochondrial axonal trafficking due to the destabilization of axonal microtubules. High concentrations of tau are associated with impaired mitochondrial distribution, potentially due to aggregates blocking axonal transport (Cheng and Bai, 2018). Tau is also associated with excessive mitochondria fragmentation, causing the mitochondria to be greater in number, yet smaller in size which is consistent with increased fission (Liu et al., 2023; Szabo et al., 2020; Ashleigh et al., 2023). Abnormalities in mitochondrial axonal trafficking, fission, fusion, and function have all been detected prior to the development of amyloid plaques or memory impairment in AD and AD models, suggesting mitochondrial dysfunction acts as a driving mechanism in disease (Praticò et al., 2001; Cheng and Bai, 2018). However, other studies suggest that misfolded tau and Aβ may instead drive mitochondrial dysfunction (Di Filippo et al., 2010; Swerdlow, 2018). It remains unclear which appears first in AD: protein aggregation or mitochondrial defects.

Studies across *in vivo, in vitro*, and postmortem settings have shown that monomeric and oligomeric Aβ interact with mitochondria and can localize within them (Manczak et al., 2006). Studies have also found that Aβ interacts with mitochondrial proteins (Pagani and Eckert, 2011; Hu et al., 2018). Increased Aβ levels and mitochondrial complex I dysfunction elevate ROS production, creating a self-perpetuating cycle of toxicity in AD models (Joh and Choi, 2017).

### 4.2 Parkinson’s disease

As in AD, mitochondrial dysfunction has been widely implicated in the development and progression of PD and is thought to be central to disease pathogenesis (Henrich et al., 2023; Grünewald et al., 2019; Monzio Compagnoni et al., 2020; Macdonald et al., 2018; Abou-Sleiman et al., 2006; Alqahtani et al., 2023). The involvement of mitochondria in PD was first suggested after MPTP and rotenone were found to inhibit complex I function in the electron transport chain (ETC), creating parkinsonian-like symptoms (Sian et al., 1999). Additional contributors to mitochondrial dysfunction in familial PD include mutations in genes involved in mitochondrial processes. These genes include parkin, *LRRK2*, *PINK1*, *DJ1*, and *SNCA* (Abou-Sleiman et al., 2006; Canet-Avilés et al., 2004; Hsu et al., 2000; Silvestri et al., 2005; Polymeropoulos et al., 1997). Alteration in mtDNA has also been observed in PD, including increased mutations, deletions, and impaired maintenance (Dölle et al., 2016).

Not only is α-syn identified as the primary constituent of LB but it may be a major contributor to mitochondrial dysfunction in PD. α-Syn has been observed to interact with mitochondrial membranes and influence mitochondrial health in PD, influencing events of fission and fusion which result in an increase of mitochondrial fragmentation (Shavali et al., 2008; Caughey and Lansbury, 2003; Krzystek et al., 2021; Li et al., 2023). Both wild type and familial PD-associated α-syn variants localize to mitochondria and induce oxidative stress in PD models (Chinta et al., 2010; Devi et al., 2008; Haque et al., 2022). Wild type α-syn, when overexpressed in neurons *in vitro*, was found to induce mitochondrial structural abnormalities and functional deficits (Nakamura, 2013). α-Syn has also been shown to interact with TOM20, an outer mitochondrial membrane protein involved in protein import, thereby inhibiting mitochondrial protein import (Needs et al., 2023). As in AD, it is unclear whether protein aggregation or mitochondrial dysfunction occurs first. Evidence suggests mitochondrial dysfunction may act as both an upstream influencer as well as a downstream consequence of α-syn aggregation (Lurette et al., 2023; Geibl et al., 2024).

### 4.3 Overlap

**FIGURE 1:**
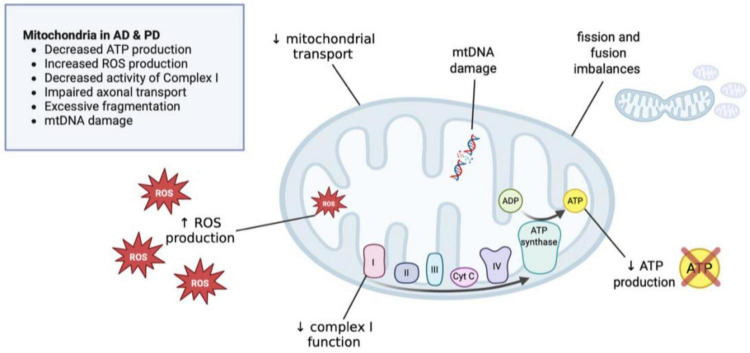
Overlapping features of mitochondrial dysfunction in Alzheimer’s disease and Parkinson’s disease. Mitochondrial dysfunction in Alzheimer’s and Parkinson’s diseases displays overlapping features including decreased ATP production, increased ROS, decreased activity in complex I of the electron transport chain, impaired axonal transport of mitochondria and other neuronal cargo, fission and fusion imbalances resulting in excessive fragmentation consistent with enhanced fission, and mtDNA damage.

Both AD and PD exhibit multiple mechanisms implicated in mitochondrial dysfunction, including mtDNA damage, increased oxidative stress, impaired mitochondrial bioenergetics, and aberrant mitochondrial dynamics (Figure 1; Gatt et al., 2016; Coskun et al., 2012; Yan et al., 2013). Physiologically, mitochondria are the primary generators of ATP via the ETC, taking place in the inner mitochondrial membrane. Consequently, ATP production can result in oxidative stress through the production of ROS, a byproduct of mitochondrial respiration that is known to increase throughout normal aging, but even more so in AD and PD contributing to their complex pathological processes (Terni et al., 2010; Manoharan et al., 2016; Watanabe et al., 2025).

Complex I of the ETC represents a key point of overlap in mitochondrial deficits between AD and PD, exhibiting reduced expression and activity in postmortem tissue from both AD and PD (Terada et al., 2021; Sian et al., 1999). A consequence of complex I impairment is increased production of ROS, which contributes to AD and PD progression (Marella et al., 2009; Joh and Choi, 2017; Bhatt et al., 2021; Dias et al., 2013). As mitochondrial dysfunction persists, mitochondrial bioenergetics further declines leading to further impairment in ATP synthesis and neuronal damage (Strope et al., 2022; Watanabe et al., 2025). The shared/convergent mitochondrial features in AD and PD are summarized in Table 1.

**TABLE 1:** Comparison of mitochondrial dysfunction mechanisms in Alzheimer’s disease and Parkinson’s disease.

Mechanism	AD	PD	Shared outcome	References
Complex I dysfunction	Reduced activity in postmortem tissue; associated with Aß/tau interactions	Inhibited by toxins in PD models (MPTP, rotenone); reduced activity in PD brains	↓ATP, ↑ ROS	(Adlimoghaddam et al., 2019; Terada et al., 2021; Sian et al., 1999; Marella et al., 2009; Joh and Choi, 2017; Bhatt et al., 2021; Dias et al., 2013; Terada et al., 2022; Gao et al., 2025; Troutwine et al., 2022)
Oxidative stress	Increased ROS from Aβ + mitochondrial dysfunction	α-syn-induced oxidative stress; ETC dysfunction	Cellular damage, feedback to protein aggregation	(Hirai et al., 2001; Area-Gomez et al., 2018; Wang et al., 2025; Marella et al., 2009; Joh and Choi, 2017; Bhatt et al., 2021; Dias et al., 2013; Terni et al., 2010; Manoharan et al., 2016; Watanabe et al., 2025)
ATP production/bioenergetics	Impaired ATP generation due to ETC dysfunction	Reduced ATP linked to mitochondrial defects	Energy failure → neuronal vulnerability	(Hirai et al., 2001; Area-Gomez et al., 2018; Wang et al., 2025; Terni et al., 2010; Manoharan et al., 2016; Watanabe et al., 2025; Strope et al., 2022)
Mitochondrial dynamics	Increased fragmentation associated with tau pathology	Altered dynamics linked to α-syn and PD genes	Structural instability of mitochondria	(Praticò et al., 2001; Cheng and Bai, 2018; Liu et al., 2023; Szabo et al., 2020; Ashleigh et al., 2023; Shavali et al., 2008; Caughey and Lansbury, 2003; Krzystek et al., 2021; Li et al., 2023)
Axonal transport	Impaired due to tau-mediated microtubule disruption	Reduced mitochondrial trafficking in neurons	Synaptic dysfunction	(Praticò et al., 2001; Cheng and Bai, 2018; Gatt et al., 2016; Coskun et al., 2012; Yan et al., 2013)
mtDNA damage	Increased mutations and oxidative damage	mtDNA deletions and impaired maintenance	Progressive mitochondrial decline	(Dölle et al., 2016; Hirai et al., 2001; Area-Gomez et al., 2018; Wang et al., 2025)
Protein-mitochondria interaction	Aß and tau interact with mitochondrial proteins	α-syn localizes to mitochondria and inhibits protein import (e.g., TOM20)	Direct disruption of mitochondrial function	(Manczak et al., 2006; Pagani and Eckert, 2011; Hu et al., 2018; Needs et al., 2023; Chinta et al., 2010; Devi et al., 2008; Haque et al., 2022)

## 5 Current treatments and therapies

### 5.1 Alzheimer’s disease

Currently, there is no cure for AD, but there are treatments that may reduce symptoms and potentially slow disease progression. The Food and Drug Administration has divided the current medications into two categories: those that temporarily ease symptoms and those that modify disease progression (Alzheimer’s Association, 2022). Symptomatic treatments include medications such as cholinesterase inhibitors and NMDA receptor antagonists (Alzheimer’s Association, 2022). These drugs aim to reduce or control cognitive and behavioral symptoms, allowing patients to maintain their abilities to perform activities of daily living. Treatment options for potentially slowing disease progression are fewer, but emerging research into anti-amyloid and anti-tau strategies are in progress. As of 2023, two potentially disease-modifying immunotherapies are FDA approved for the treatment of mild AD. These two drugs, Donanemab and Lecanemab, are anti-amyloid antibody intravenous infusion therapies that work by removing beta-amyloid from the brain (Alzheimer’s Association, 2022). The goal of these drugs is to address an underlying pathology of AD rather than symptomology.

### 5.2 Parkinson’s disease

The gold standard treatment for PD motor symptoms is levodopa, which is the precursor to DA (Gandhi and Saadabadi, 2023; Tambasco et al., 2018). By taking levodopa, DA can be replaced in surviving SN neurons, but this is not a cure since dopaminergic neurons continue to degenerate. Levodopa is often combined with carbidopa to aid its ability to reach the brain by blocking peripheral DA metabolism (Mayo Clinic, 2023). DA agonists are also used as therapy; however, instead of directly boosting DA levels, these drugs mimic the effects of DA in the brain (Mayo Clinic, 2023). Monoamine oxidase B inhibitors and Catechol O-methyltransferase inhibitors are also commonly used, helping to block the breakdown of DA (Mayo Clinic, 2023). A non-drug-based treatment option with positive results is deep brain stimulation (DBS). DBS targets nodes within the basal ganglia–thalamic circuitry, with stimulation of the thalamus improving tremor, the globus pallidus improving rigidity and dyskinesia, and the subthalamic nucleus improving tremor, akinesia, and rigidity (Hariz and Blomstedt, 2022).

### 5.3 Overlap

Used to treat both AD and PD, cholinesterase inhibitors target memory impairment resulting from the loss of cholinergic neurons in the BF; however these may enhance PD motor symptoms (Alzheimer’s Association, 2022). Additionally, both AD and PD treatment options include memantine, an NMDA receptor agonist, to manage cognitive symptoms by protecting the brain from excess glutamate (Olivares et al., 2012). Neither AD nor PD currently have an available cure, and mitochondria-targeting drugs are not yet in clinical use. Further investigation of shared mechanisms, including protein co-pathology and mitochondrial dysfunction, may enable the development of more effective therapies for both AD and PD.

## 6 Mitochondria-specific therapies in development

### 6.1 Mitoquinone

As previously described, mitochondrial dysfunction is a well-established feature of both AD and PD. Accordingly, multiple therapeutic strategies are in development to target mitochondrial pathways, but none have yet surpassed the preclinical phase. One of the most extensively explored strategies for targeting mitochondrial dysfunction in neurodegenerative disease employs antioxidants in an attempt to mitigate oxidative stress (Macdonald et al., 2018). While ROS have been discussed as a key downstream consequence of mitochondrial impairment, conventional antioxidants have shown limited efficacy, in part due to their inability to accumulate within mitochondria at therapeutically relevant concentrations (Fields et al., 2023). To address this limitation, mitochondria-targeted antioxidants (MTAs) have been developed to selectively localize within the organelle. Mitoquinone (MitoQ) is one of the most well-characterized compounds in this class. MitoQ consists of an ubiquinone moiety, analogous to coenzyme Q10, linked to a lipophilic triphenylphosphonium (TPP^+^), which enables selective accumulation within mitochondria via mitochondrial membrane potential, whereas coenzyme Q10 on its own does not. Once localized, MitoQ converts to its active antioxidant form, ubiquinol, where it functions to scavenge and neutralize ROS to aid in limiting oxidative damage (Almikhlafi et al., 2023). Animal studies have demonstrated that MitoQ can reduce oxidative stress, improve mitochondrial function, and mitigate neurodegenerative phenotypes in both AD and PD models. However, despite strong preclinical support and confirmed safety at chronic, high doses, clinical outcomes have been more variable (Shinn and Lagalwar, 2021; Choi et al., 2024).

### 6.2 Ubiquitin-specific protease 30

While antioxidant approaches aim to mitigate downstream consequences of mitochondrial dysfunction, an alternative strategy is to enhance mitochondrial quality control by promoting removal of the impaired mitochondria. Mitophagy, a mitochondria-specific form of autophagy, plays a critical role in maintaining mitochondrial integrity, and its disruption has been strongly implicated in both AD and PD. In particular, the PINK1–Parkin pathway has emerged as a central regulator of mitophagy, where damaged mitochondria are tagged for degradation through ubiquitination of outer mitochondrial membrane (Bingol et al., 2014). This pathway is negatively regulated through ubiquitin-specific protease 30 (USP30), a mitochondrial deubiquitinase found to localize to the outer mitochondrial membrane and function by removing ubiquitin from Parkin-tagged mitochondria. As such, inhibition of USP30 has emerged as a promising therapeutic strategy to enhance mitophagy and facilitate the clearance of dysfunctional mitochondria (Wang et al., 2022). Cell and animal models of AD and PD have found inhibition of UPS30 to enhance mitophagy, improve mitochondrial function, reduce oxidative stress, and even provide neuroprotection (Jiang et al., 2023; Fang et al., 2023; Siwach et al., 2025). Notably, mitophagy-enhancing strategies, including USP30 inhibition, have advanced to early-phase clinical trials, underscoring the potential of targeting mitochondrial quality control pathways in neurodegenerative disease such as AD and PD (Antico et al., 2025).

### 6.3 Influence on tau and α-synuclein pathologies

The relevance of these therapeutic strategies is further underscored by the established links between tau and α-syn pathologies and mitochondrial dysfunction. As discussed earlier, both tau and α-syn have been shown to impair mitochondrial function through distinct but overlapping mechanisms (Table 1). In addition, elevated mitochondrial ROS has been associated with both tau hyperphosphorylation and α-syn aggregation, suggesting that oxidative stress may not only be a consequence of pathology but also a driver of its progression. In this context, mitochondria-targeted antioxidants such as MitoQ may serve to interrupt this feedforward cycle by reducing ROS-mediated damage and limiting downstream effects on protein aggregation.

In parallel, growing evidence indicates that both tau and α-syn contribute to impaired mitochondrial quality control, leading to the accumulation of dysfunctional mitochondria (Antico et al., 2025). Disruption of mitophagy pathways has been observed in models of tau and α-syn pathology, further exacerbating cellular stress and neuronal vulnerability (Jiang et al., 2023; Fang et al., 2023; Siwach et al., 2025). Enhancing mitophagy through inhibition of negative regulators such as USP30 therefore represents a strategy to restore mitochondrial turnover and energetic balance that in turn may fuel cellular defense mechanisms to reduce protein aggregate burden. Together, this framework positions mitochondrial dysfunction not only as a downstream consequence of tau and α-syn toxicity, but as an active contributor to disease progression, and highlight mitochondria-targeted interventions as a means to disrupt this pathogenic feedback loop.

## 7 Concluding remarks

**FIGURE 2:**
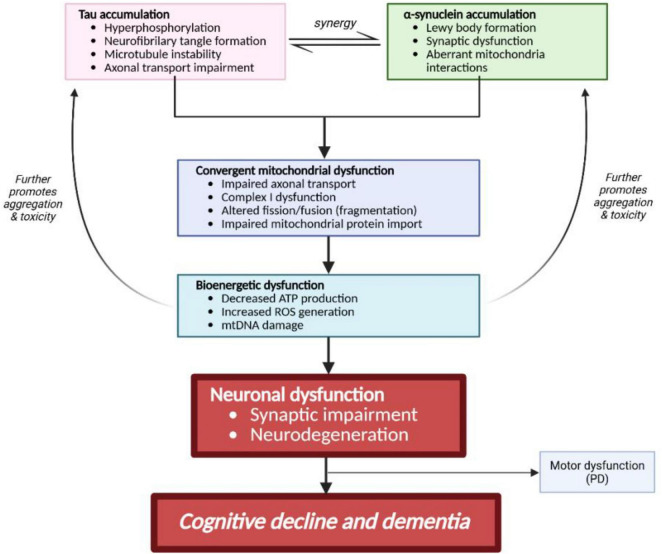
Convergent model of tau and α-synuclein–mediated mitochondrial dysfunction driving cognitive decline. Intracellular tau and α-synuclein interact synergistically and converge on mitochondrial pathways, disrupting key processes including axonal transport, complex I activity, mitochondrial dynamics, and protein import. These alterations impair mitochondrial bioenergetics, resulting in reduced ATP production, increased reactive oxygen species, and mitochondrial DNA damage. Ultimately these metabolic disturbances contribute to neuronal dysfunction and degeneration, driving cognitive decline and dementia. Feedback interactions between mitochondrial dysfunction and protein pathology may further exacerbate disease progression. While Parkinson’s disease is initially characterized by motor dysfunction, these shared mechanisms also contribute to cognitive impairment as the disease advances.

While AD and PD have historically been defined by their distinct initial clinical presentations, both diseases eventually involve cognitive decline and dementia. Significant overlap exists due to the prevalence of mixed pathologies and shared regions that undergo neurodegeneration. Mixed pathologies are common in late-onset neurodegeneration, with tau and α-syn frequently co-existing, exacerbating neuronal dysfunction and enhancing cognitive decline when both proteinopathies are present. Tau and α-syn pathologies are both known to impact mitochondrial function and cause oxidative stress through mechanisms such as complex I dysfunction, altered mitochondrial dynamics and bioenergetics, and impaired axonal transport (Figure 2). A comparison of the clinical, pathological, and mitochondrial features of AD and PD is summarized in Table 2.

**TABLE 2:** Comparative overview of clinical features, pathological hallmarks, and mitochondrial dysfunction in Alzheimer’s disease and Parkinson’s disease.

Disease	AD	PD
Clinical features		
Primary clinical presentation	Progressive memory loss and cognitive decline	Motor dysfunction (bradykinesia, rigidity, tremor)
Cognitive impairment	Early and prominent	Typically later-stage
Disease progression	Gradual cognitive decline leading to dementia	Motor symptoms first, cognitive decline in later stages
Pathological hallmarks		
Key protein aggregates	Amyloid-β plaques; tau (neurofibrillary tangles)	α-synuclein (Lewy bodies)
Primary affected region	Hippocampus	Substantia nigra
Protein co-pathology	Tau frequently co-occurs with α-syn	α-syn often co-occurs with tau
Major co-affected region	Basal forebrain	
Mitochondrial dysfunction		
Complex I activity	Reduced	Reduced
Oxidative stress	Increased	Increased
ATP production	Decreased	Decreased
Mitochondrial dynamics	Increased fragmentation	Increased fragmentation
Axonal transport	Impaired (tau-related)	Impaired (α-syn-related)
mtDNA damage	Increased	Increased

Shared mitochondrial dysfunction contributes to neuronal degeneration and cognitive decline.

Currently, there are no FDA-approved mitochondria-targeting treatments for either AD or PD. Given the extensive evidence demonstrating mitochondrial dysfunction as a driver of disease in both AD and PD, the development of therapies targeting mitochondria could serve as a unifying approach to mitigate neurodegeneration and cognitive decline. However, given the multifactorial nature of neurodegenerative disease, targeting mitochondrial dysfunction in isolation may not be sufficient to fully halt disease progression. As such, there is increasing interest in combining mitochondria-targeted strategies with existing therapeutic approaches. Current treatments for AD and PD largely focus on symptomatic management or modulation of specific pathways, such as cholinergic signaling, dopaminergic function, or protein pathologies. However, these approaches do not directly address the underlying mitochondrial dysfunction that contributes to neuronal vulnerability. Integrating mitochondria-targeted interventions, including antioxidants such as MitoQ or mitophagy enhancers such as USP30 inhibitors, with existing therapies may provide a more comprehensive approach by simultaneously targeting both upstream drivers and downstream consequences of disease. For example, combining mitochondria-targeted strategies with therapies aimed at reducing tau or α-syn burden may help to disrupt the reciprocal relationship between protein aggregation and mitochondrial dysfunction. In this context, mitochondrial interventions may enhance neuronal resilience and improve the overall efficacy of current treatments.

Ultimately, a combinatorial therapeutic approach that addresses multiple interconnected aspects of neurodegeneration, including protein aggregation and mitochondrial dysfunction, may be required to achieve impactful and sustained benefits in both AD and PD contexts.
