# Supplementary figures and images for: Toward common treatment strategies: convergent proteinopathies and mitochondrial dysfunction in Alzheimer’s and Parkinson’s diseases
Source: Front Neurosci. 2026 May 26;20:1846384. doi: 10.3389/fnins.2026.1846384 (PMC13246617; doi:10.3389/fnins.2026.1846384)

**Supplementary File 1:**

Initial (Left) and Final (Right) Generative AI Prompts.


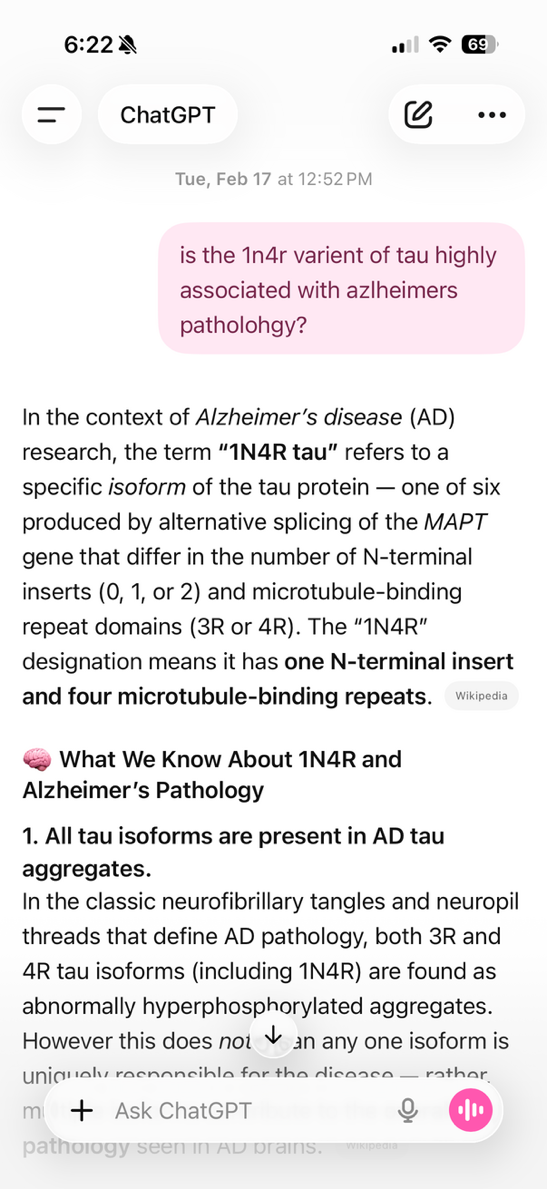

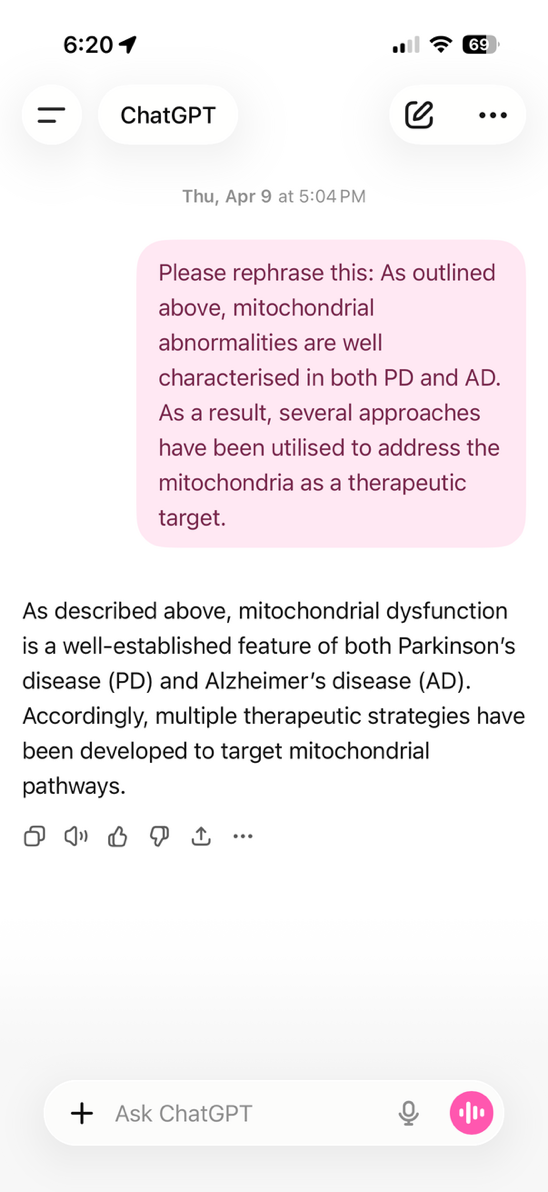

Supplement: Supplementary file 1 [file Data_Sheet_1.docx]
